# Supplementary material for: Deletion of the Candida albicans TLO gene family using CRISPR-Cas9 mutagenesis allows characterisation of functional differences in α-, β- and γ- TLO gene function
Source: PLoS Genet. 2023 Dec 4;19(12):e1011082. doi: 10.1371/journal.pgen.1011082 (PMC10721199; doi:10.1371/journal.pgen.1011082)
Supplement: S1 Text — (PDF) [file pgen.1011082.s001.pdf]

Generation of MAY1244, a chromosome 5 (Chr5) AB disomic derivative of AHY940

MAY1244 is a chromosome 5 (Chr5) AB disomic derivative ofAHY940 – an SC5314 *LEU2* heterozygous strain that is Chr5 ABB trisomic – sent from the Hernday laboratory. MAY1244 was generated by streaking MAY1035 onto solid yeast extract-peptone-dextrose (YPD) medium from -80°C freezer stocks and incubating overnight at 30°C. Cells from the thick part of the streak were resuspended in 1X PBS and replated to fresh solid YPD medium to grow up isolated colonies. These plates were then incubated for one day at 30°C, then for six days at room temperature. Colonies were then inspected for wrinkling, which indicated Chr5 trisomy, and six non-wrinkling colonies were frozen down together in 500 µL YPD + 500 µL 50% glycerol as MAY1244.

The Chr5 configuration of MAY1244 was initially investigated by assessing the presence of the *MTL* (mating type-like) locus via colony PCR. To test for each *MTL* idiomorph, primers targeting *PAP**a*** (Chr5 A; ALO73 = 5'-CGGGTAAAATATGGTCGC-3', ALO74 = 5'-TACTTTCCACCATCCCACT-3') and *PAPα* (Chr5 B; ALO75 = 5'-CTGGCATTCGATGAAGTCTA-3', ALO76 = 5'-CATGTCCGATTCAATGGCCC-3') were used to define *MTL***a** and *MTL*α, respectively. Strains were assayed twice independently alongside strains P60002 (*MTL***a**/**a**) and 19F (*MTL*α/α) as controls.

To identify the relative copy number of the Chr5 A and B homologs, genomic DNA (gDNA) was extracted from an overnight culture of MAY1244, MAY1 (wildtype SC5314), and MAY1035 using the MasterPure™ Yeast DNA Purification Kit (VWR) with the optional RNase A treatment. gDNA was also extracted from three isolated colonies of MAY1244 to check subpopulations. gDNA concentrations were quantified using the Qubit dsDNA Broad Range Assay Kit (ThermoFisher), and a region spanning Chr5: nucleotides 929387-929763 (Assembly 21 [2, 3]) was amplified by PCR with oligos **1458** = 5'-TCAATACGCAACTTCCCAGTT-3' and **1459** = 5'-TTCAACCTTTCCCAGAATGTC-3'), which were based on oligos used for the SNP-RFLP marker 2093/2390 reported in Forche *et al.* [4] This region has 7 heterozygous positions in SC5314 that could be used to distinguish between homologs A and B based on relative peak heights for relative copy number estimates of each homolog. Sequencing was conducted by the Genomics Shared Resource at The Ohio State University and was supported by the Cancer Center Support Grant CCSG: P30CA016058 (Sanger sequencing, Genomics Shared Resource at The Ohio State University)

Relative chromosome copy number was determined using a previously developed multiplexed PCR method from Arbour *et al.* [5] Multiplex PCR reactions were performed using the QIAGEN Multiplex PCR Kit (Qiagen) as described in Arbour *et al. [5]*, with gDNA for MAY1244, MAY1, and MAY1035 extracted as written above. Briefly, 50 µL reactions had final concentrations of 1X QIAGEN Multiplex PCR Master Mix, 0.125 µM equimolar oligos for either A (left arm) or B (right arm) oligo sets, and 1 ng/µL gDNA (50 ng input per 50 µL reaction). PCRs were performed in an Applied Biosystems ProFlex PCR System using the cycling conditions as described in Arbour *et al.* [5]. Multiplex PCR reaction yields were quantified using the Qubit dsDNA Broad Range Assay Kit. The multiplex PCR reactions were diluted to 1000 pg/µL with ultrapure water, loaded onto an

Agilent High Sensitivity DNA Kit (Agilent,) chip, and ran using an Agilent 2100 Bioanalyzer (Agilent). Bioanalyzer traces of MAY1244 were overlaid with MAY1 (Chr5 AB) and MAY1035 (Chr5 ABB) traces to verify that MAY1244 was diploid.

To confirm MAY1244 ploidy and to screen for loss of heterozygosity (LOH) or major genomic rearrangements that may have occurred during the construction of AHY940 or generation of MAY1244, whole genome sequencing was performed for MAY1244 using Illumina short-read technology. gDNA was extracted from an overnight culture of MAY1244 using the Zymogen *Quick*-DNA Fungal/Bacterial Miniprep Kit (Zymogen). gDNA concentration was quantified using the Qubit dsDNA Broad Range Assay Kit. Following quantification, the sample was sent to the Applied Microbiology Services Lab (AMSL) at The Ohio State University for processing. Libraries were constructed via tagmentation and dual index barcoding using a modified protocol for the Illumina (L) Tagmentation Kit (Illumina) to produce average final fragment sizes of approximately 450-500 bp. The library was sequenced for 2×150 paired-end reads on an Illumina NextSeq 2000. Reads were demultiplexed and Illumina adaptors were trimmed by AMSL. Read quality was assessed using FastQC (v0.11.7) [6] and low-quality positions were trimmed using Trimmomatic (v0.35 LEADING:20 TRAILING:20 SLIDINGWINDOW:4:20 MINLEN:35) [7], after which the trimmed data was checked again using FastQC. Reads were mapped to *Candida albicans* reference genome Assembly 21 (A21-s02-m09-r10) – obtained March 2, 2021 from the *Candida* Genome Database website (http://www.candidagenome.org/download/sequence/C_albicans_SC5314/Assembly21/current/ C_albicans_SC5314_A21_current_chromosomes.fasta.gz, [3]) – using Bowtie 2 (v2.2.6-2) [8] with parameters "-3 1" to improve downstream analysis. Samtools (v0.1.19) [9] was then used to generate .bam files, read sorting, and sample indexing. Read alignment quality was interrogated via visual scanning using IGV (Integrated Genome Viewer, v2.9.2) [10, 11] for aneuploidy, LOH, and major genomic rearrangements. Secondary checks for ploidy were performed using BBmap (v39.01, Bushnell B. - sourceforge.net/projects/bbmap/) to generate a chromosome pileup file (calculating coverage across all chromosomes). Sequence data is available for download from the NCBI sequence read archive, BioProject no. PRJNA962819.

Analysis of Chromosome structure in the *TLO* mutants

Simultaneous induction of large-scale double-strand breaks required to construct the *tlo*Δ strains necessitated karyotypic analysis to identify potential changes in chromosomal copy number and/or rearrangements. Using short read sequencing, we compared each *tlo*Δ strain to the parental CRISPR competent strain, AHY940. In the CC10 mutant selected for further analysis, a single loss of heterozygosity (LOH) event was detected on the extreme left end of chromosome 6 (Chr6) approximately 55 kb long. The other mutant selected, CC16, did not show any new large LOHs compared to the parent. Importantly, both *tlo*Δ strains resolved the trisomic Chr5 ABB karyotype to become disomic for Chr5, retaining one of each homolog A and B. Read depth

indicated all other chromosomes remained disomic, although, CHEF and long read sequencing revealed novel chromosomes in both strains.

The *tlo*Δ CC10 mutant contained major rearrangements involving multiple chromosomes (Fig. S3). A truncation arose in the left arm of one copy of Chr1 (Chr1L) that began in the 3′ coding sequence of *TLOα34*, producing a new telomere for the left arm within *TLOα34's* coding sequence. One of the remaining Chr1L fragments containing the sequence between *TLOα3* and *TLOα34* fused with Chr7. Long read sequencing showed that the introduced CRISPR system recombined with the 3′ coding sequence of *TLOα3* on the 1L fragment and *TLOγ16* in the subtelomere of Chr7R to produce the fusion product. Both Chr1L fragments are fused on the centromeric side to either Chr4L at the *TLOα9* coding sequence or Chr5L at the *TLO*α-like sequence located downstream of *TLOγ11*. We were unable to resolve if the Chr1L fragment fused to Chr7 is connected to Chr4 or Chr5 due to the distance between each fusion event, and the similar size of these alternate fusion products by CHEF gel.

The second *tlo*Δ strain, CC16, contained fewer chromosomal rearrangements than CC10 (Fig. S3). The only major rearrangements occurred between Chr1 and Chr7. A single Chr1 homolog was truncated as in CC10, with a telomeric sequence capping the truncation product at *TLOα34* on Chr1L, whereas the second Chr1 homolog was entirely intact. Yet, the intact copy of Chr1 contained an inversion of nearly two-thirds of the chromosome beginning at the *TLOα34* locus through Chr1R, including the centromere. This inversion involved recombination between the Rho long terminal repeat (LTR) telomeric of *TLOα34* on Chr1L fragment and the Rho LTR located centromeric of *TLOγ4* on Chr1R*.* The remaining Chr1L fragment from the truncated homolog fused to one Chr7 homolog via homologous sequence between the same Rho LTR telomeric of *TLOα34* and the partial Rho LTR centromeric of *TLOγ16*.

Sequence data is available for download from the NCBI sequence read archive, BioProject no. PRJNA962819.

References

1. Nguyen N, Quail MMF, Hernday AD. An efficient, rapid, and recyclable system for CRISPR-mediated genome editing in *Candida albicans*. mSphere. 2017;2: e00149-17 . doi: 10.1128/mSphereDirect.00149-17.

2. van het Hoog M, Rast TJ, Martchenko M, Grindle S, Dignard D, Hogues H, *et al.* Assembly of the *Candida albicans* genome into sixteen supercontigs aligned on the eight chromosomes. Genome Biol. 2007;8: R52. doi: 10.1186/gb-2007-8-4-r52.

3. Skrzypek MS, Binkley J, Binkley G, Miyasato SR, Simison M, Sherlock G. The *Candida* Genome Database (CGD): Incorporation of Assembly 22, systematic identifiers and visualization of high throughput sequencing data. Nucleic Acids Res. 2017;45: D592-D596. doi: 10.1093/nar/gkw924.

4. Forche A, Steinbach M, Berman J. Efficient and rapid identification of *Candida albicans* allelic status using SNP-RFLP. FEMS Yeast Res. 2009;9:1061-1069. doi: 10.1111/j.1567-1364.2009.00542.x.

5. Arbour M, Epp E, Hogues H, Sellam A, Lacroix C, Rauceo J, *et al.* Widespread occurrence of chromosomal aneuploidy following the routine production of *Candida albicans* mutants. FEMS Yeast Res. 2009;9: 1070-1077. doi: 10.1111/j.1567-1364.2009.00563.x.

6. Wingett SW, Andrews S. FastQ Screen: A tool for multi-genome mapping and quality control. F1000Res. 2018; 7:1 338. doi: 10.12688/f1000research.15931.2.

7. Bolger AM, Lohse M, Usadel B. Trimmomatic: a flexible trimmer for Illumina sequence data. Bioinformatics. 2014;30: 2114-2120. doi: 10.1093/bioinformatics/btu170.

8. Langmead B, Salzberg SL. Fast gapped-read alignment with Bowtie 2. Nat Methods. 2012;9: 357-359. doi: 10.1038/nmeth.1923.

9. Danecek P, Bonfield JK, Liddle J, Marshall J, Ohan V, Pollard MO, *et al.* Twelve years of SAMtools and BCFtools. GigaScience. 2021;10:.giab008. doi: 10.1093/gigascience/giab008.

10. Robinson JT, Thorvaldsdottir H, Winckler W, Guttman M, Lander ES, Getz G, *et al.* Integrative genomics viewer. Nat Biotechnol. 2011;29: 24-26. doi: 10.1038/nbt.1754.

11. Thorvaldsdottir H, Robinson JT, Mesirov JP. Integrative Genomics Viewer (IGV): High-performance genomics data visualization and exploration. Brief Bioinform. 2013;14:178-192. doi: 10.1093/bib/bbs017.

12. Schwartz DC, Cantor CR. Separation of yeast chromosome-sized DNAs by pulsed field gradient gel electrophoresis. Cell. 1984;37: 67-75. doi: 10.1016/0092-8674(84)90301-5.
