# Supplementary material for: Deletion of the Candida albicans TLO gene family using CRISPR-Cas9 mutagenesis allows characterisation of functional differences in α-, β- and γ- TLO gene function
Source: PLoS Genet. 2023 Dec 4;19(12):e1011082. doi: 10.1371/journal.pgen.1011082 (PMC10721199; doi:10.1371/journal.pgen.1011082)
Supplement: S1 Fig — (PDF) [file pgen.1011082.s002.pdf]

Figure S1

A

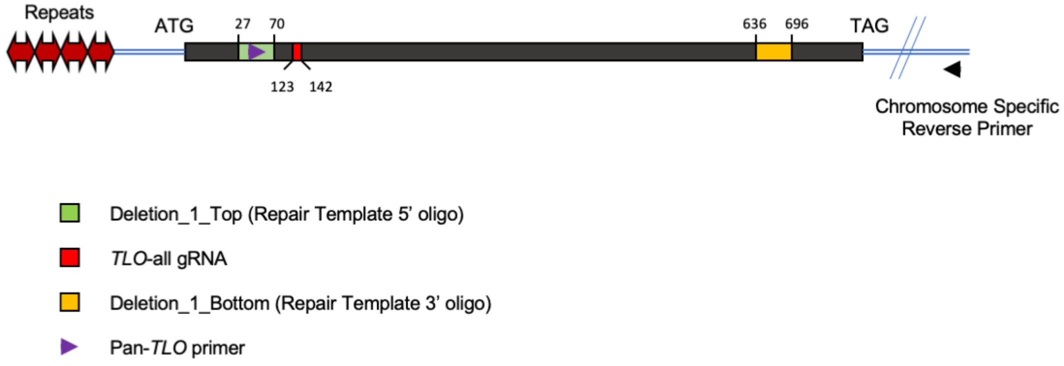

B

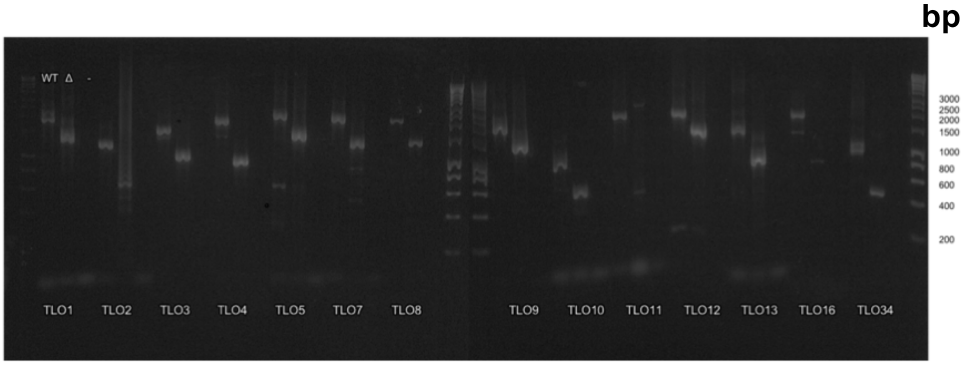

Figure S1. (A) Diagram of the *TLOα1* gene locus outlining the CRISPR-Cas9 deletion strategy used in the current study. The guide RNA designed to target all *TLO* genes corresponds to a conserved region found at position 123-142 in *TLOα1* (Table S2). The repair template was constructed using overlapping oligonucleotides homologous to regions in the 5´ and 3´ ends of the *TLO* ORF corresponding to positions 27-70 and 636-696 of *TLOα1.* (B) Screening of *TLO* mutants was carried out using a Pan-*TLO* primer and a primer specific to each chromosome (Table S2). Agarose gel shows the results of PCR screening of the *tlo*Δ mutant CC16 with Pan-*TLO* and chromosome-specific primers showing truncation at each locus. Each lane is labeled alternately as WT (AHY940) or Δ (CC16).
