# Supplementary material for: Deletion of the Candida albicans TLO gene family using CRISPR-Cas9 mutagenesis allows characterisation of functional differences in α-, β- and γ- TLO gene function
Source: PLoS Genet. 2023 Dec 4;19(12):e1011082. doi: 10.1371/journal.pgen.1011082 (PMC10721199; doi:10.1371/journal.pgen.1011082)
Supplement: S2 Fig — (PDF) [file pgen.1011082.s003.pdf]

**Figure S2**

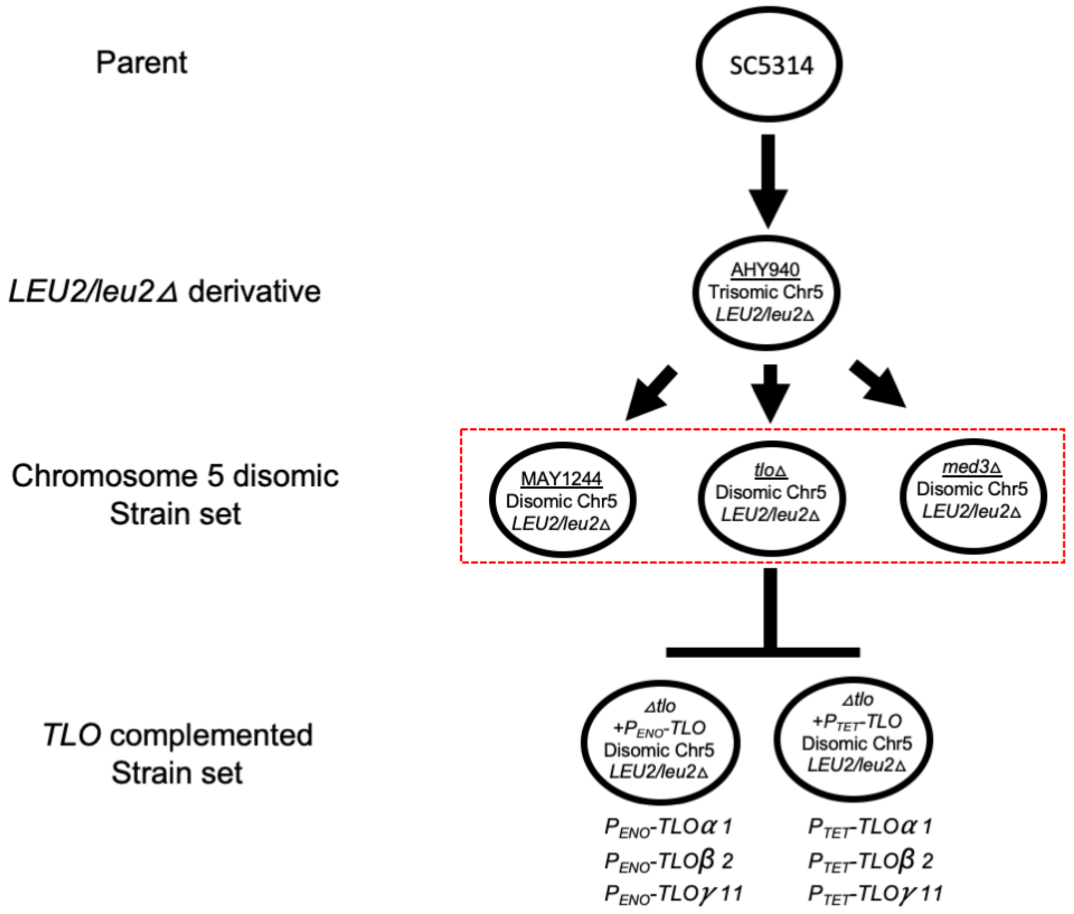


**Figure S2. Lineage of the strains derived in this study (full genotypes in Table S1).** AHY940 is a *LEU2/Leu2*Δ derivative of *C. albicans* SC5314 generated in the Hernday laboratory [1]. Our analysis in this study identified that AHY940 is trisomic for chromosome 5 (ABB). The *tlo*Δ and *med3*Δ mutants generated in this study are derived from AHY940 and are spontaneous Chr5 disomic AB derivatives.

For the RNA-Seq experiments comparing the transcriptomes of the *tlo*Δ and wild type, we utilized MAY1244 which is a Chr5 disomic AB derivative of AHY940 generated in the Anderson laboratory (this study, described below).
