# Supplementary material for: Deletion of the Candida albicans TLO gene family using CRISPR-Cas9 mutagenesis allows characterisation of functional differences in α-, β- and γ- TLO gene function
Source: PLoS Genet. 2023 Dec 4;19(12):e1011082. doi: 10.1371/journal.pgen.1011082 (PMC10721199; doi:10.1371/journal.pgen.1011082)
Supplement: S3 Fig — (PDF) [file pgen.1011082.s004.pdf]

Figure S3

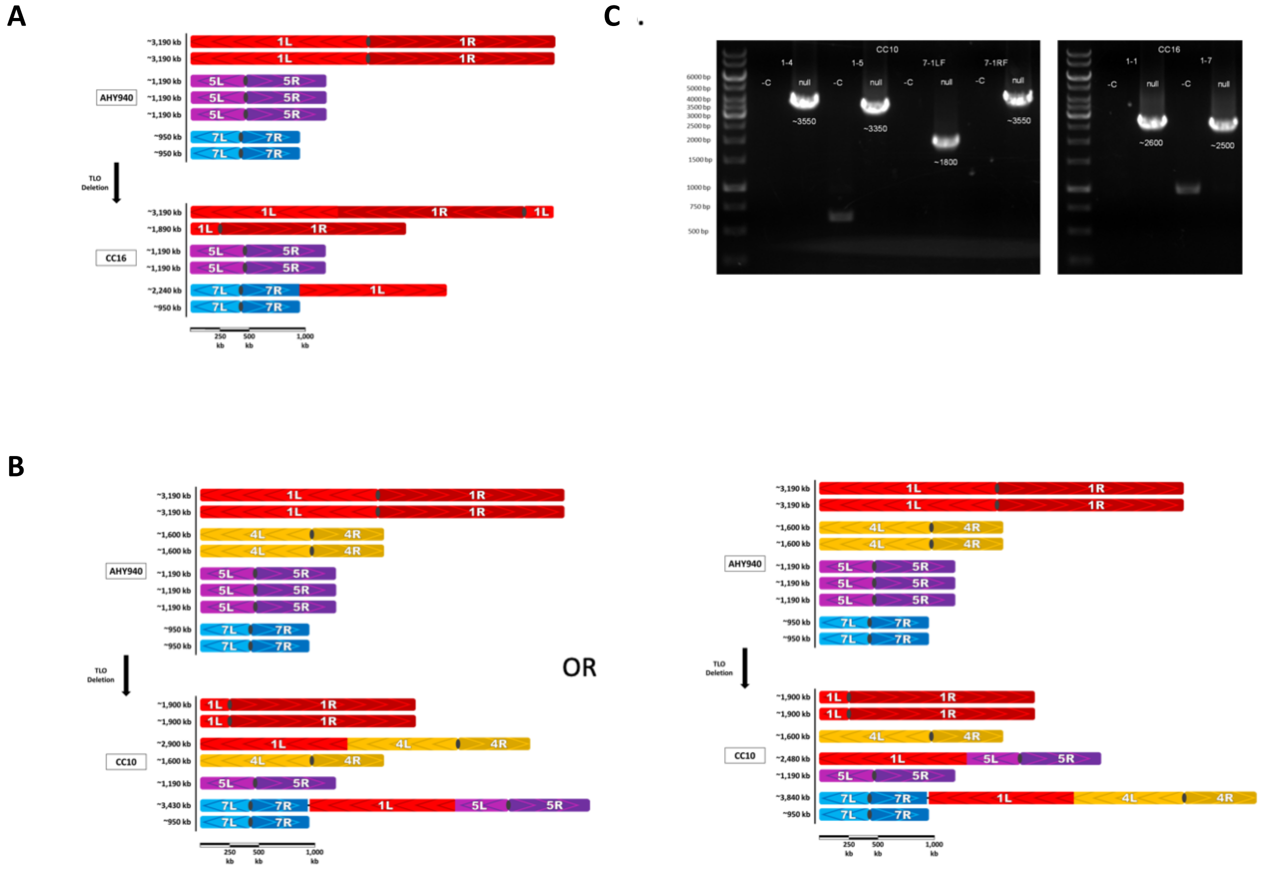

Figure S3. Karyotypes of *tlo*Δ mutants CC10 and CC16. Genomic maps represent the chromosomal rearrangements identified in the mutant *tlo*Δ strains, CC16 (A) and CC10 (B). CC10 has two possible genomic configurations involving fusions of either Chr4 or Chr5 to Chr1L or a Chr7-1L fusion product. Each chromosome is color coded, and arrows point towards chromosome arm telomeres based on the SC5314 reference genome. Black lines in CC10 represent the addition of CRISPR-Cas9 system DNA. (C) PCR checks of chromosomal fusion events in CC10 and CC16. Expected sizes of the fusion products are indicated below and each band in the null lane. The Chr7 and Chr1 fusion in CC10 is split into a left flank (7-1F) and a right flank (7-1RF) check due to the large size of the added CRISPR-Cas9 DNA. Negative control (-C) indicates the CRISPR competent parental strain.
