# Supplementary material for: Deletion of the Candida albicans TLO gene family using CRISPR-Cas9 mutagenesis allows characterisation of functional differences in α-, β- and γ- TLO gene function
Source: PLoS Genet. 2023 Dec 4;19(12):e1011082. doi: 10.1371/journal.pgen.1011082 (PMC10721199; doi:10.1371/journal.pgen.1011082)
Supplement: S4 Fig — (PDF) [file pgen.1011082.s005.pdf]

Figure S4

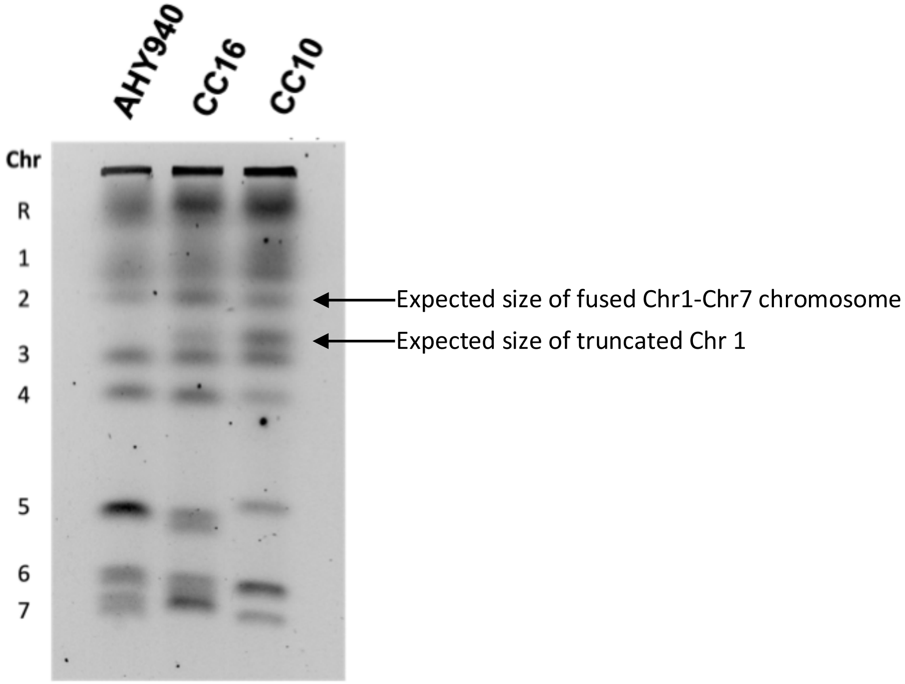

Figure S4. Visualization of karyotype by contour-clamped homogeneous electric field (CHEF) [12]. Electrophoresis of chromosomal DNA in strain AHY940 and *tlo*Δ strains CC16 and CC10. Arrows indicate the position of the predicted chromosome 1 variants identified in DNA sequence analysis including the Chr1-Chr7 fusion in CC16 (same predicted size as Chr 2) and the Chr 1 truncation (see figure S3).
