# Supplementary material for: Deletion of the Candida albicans TLO gene family using CRISPR-Cas9 mutagenesis allows characterisation of functional differences in α-, β- and γ- TLO gene function
Source: PLoS Genet. 2023 Dec 4;19(12):e1011082. doi: 10.1371/journal.pgen.1011082 (PMC10721199; doi:10.1371/journal.pgen.1011082)
Supplement: S5 Fig — (PDF) [file pgen.1011082.s006.pdf]

Figure S5

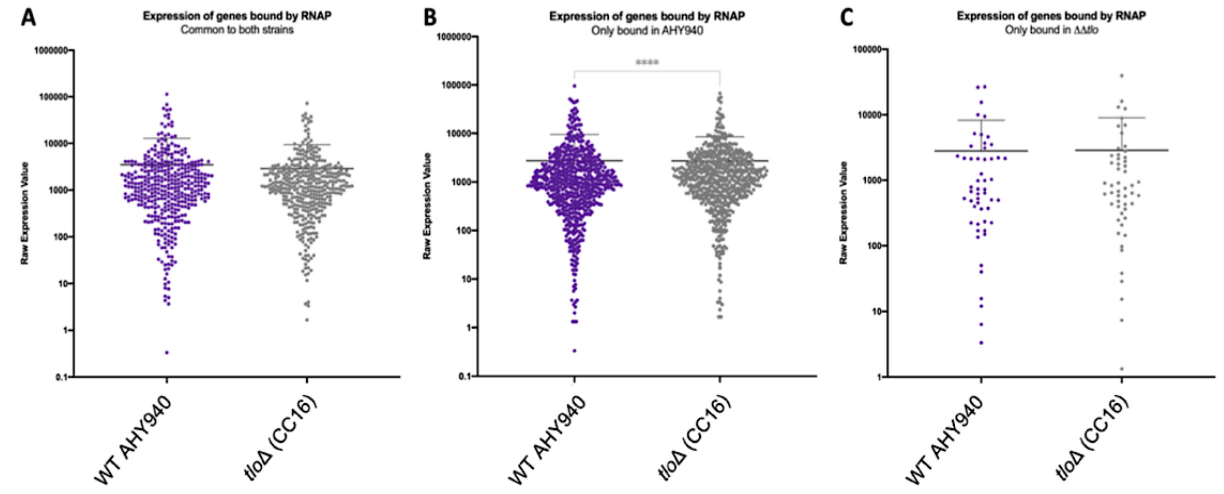

Figure S5. Expression of genes bound by RNAP II in AHY940 and the *tloΔ* mutant. The raw expression values of genes (generated in Strand NGS) in AHY940 and in the *tloΔ* mutant were compared, including genes bound by RNAP II in both strains (A, n=441), in AHY940 only (B, n=697) and in the *tloΔ* mutant only (C, n=61). Wilcoxon matched-pairs signed rank tests were performed to determine significant difference between groups. Asterisks denote significant difference (****=p <0.001).
