# Supplementary material for: Deletion of the Candida albicans TLO gene family using CRISPR-Cas9 mutagenesis allows characterisation of functional differences in α-, β- and γ- TLO gene function
Source: PLoS Genet. 2023 Dec 4;19(12):e1011082. doi: 10.1371/journal.pgen.1011082 (PMC10721199; doi:10.1371/journal.pgen.1011082)
Supplement: S6 Fig — (PDF) [file pgen.1011082.s007.pdf]

Figure S6

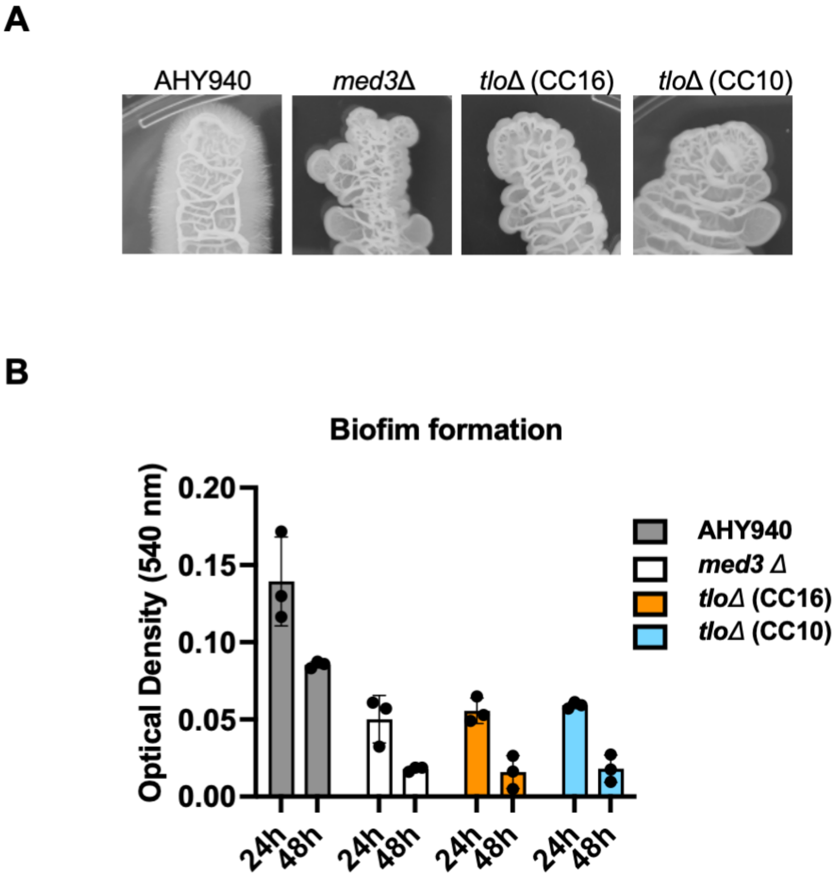

Figure S6. Phenotypes of *med3*Δ and *tlo*Δ strains CC16 and CC10 on Spider medium. (A) Morphology of strains incubated on solid Spider medium for 5 days at 30˚C. (B) Biofilm formation on 96-well tissue culture dishes (Greiner) following incubation in Spider medium at 37 ˚C. Quantification was carried out with crystal violet as described in the methods.
