# Supplementary material for: Deletion of the Candida albicans TLO gene family using CRISPR-Cas9 mutagenesis allows characterisation of functional differences in α-, β- and γ- TLO gene function
Source: PLoS Genet. 2023 Dec 4;19(12):e1011082. doi: 10.1371/journal.pgen.1011082 (PMC10721199; doi:10.1371/journal.pgen.1011082)
Supplement: S7 Fig — (PDF) [file pgen.1011082.s008.pdf]

Figure S7

A
B

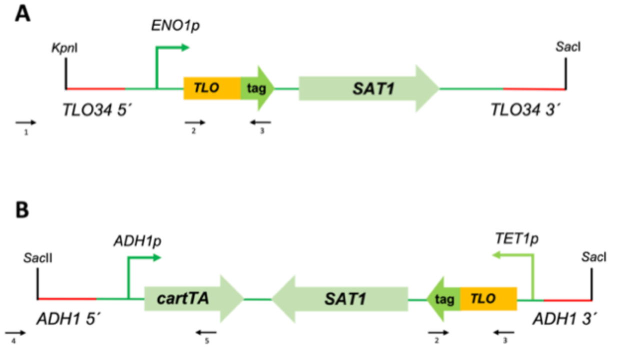

C

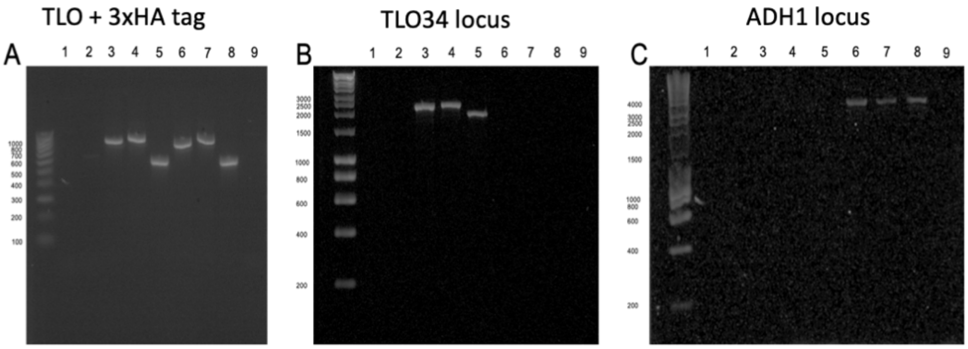

Figure S7. Constructs used to complement the *tlo*Δ strain CC16. (A) Cassette used for *TLO* gene expression with a 3xHA tag under the *ENO1* promoter (high expression) based on the SAT1-flipper [1]. (B) The *TLO* gene expression cassette using a tetracycline inducible promoter, *TET1*, described by Park and Morschhauser [2]. Black numbered arrows indicate primer binding sites for confirmatory PCRs for integration of cassettes into correct sites. Primers (Table S1) are as follows: 1) TLO34 locus F; 2) TLO (*Sal*I) F; 3) TLO tag (*Bgl*II) R; 4) ADH1 locus F; 5) carTA R.

(C) PCR confirmation of integration of the *TLO* constructs into the *tlo*Δ background. Lanes in all gels are as follows: 1) AHY940 WT; 2) *tlo*Δ; 3) P_{ENO1}-*TLOα1*; 4) P_{ENO1}-*TLOβ2*; 5) P_{ENO1}-*TLOγ11*; 6) P_{TET1}-*TLOα1*; 7) P_{TET1}-*TLO*β*2*; 8) P_{TET1}-*TLOγ11*; 9) negative control (no DNA template). (A) PCR with oligonucleotide primers “TLO (*Sal*I) F” and “TLO tag (*Bgl*II) R” targeting the HA-tagged *TLO* gene. Predicted sizes: *TLOα1* = 844 bp; *TLO*β*2* = 913 bp; *TLOγ11* = 601 bp. (B) PCR to detect reintegration of the P_{ENO1}-*TLOs* at the *TLOα34* locus using primers “TLO34 locus F” and “TLO tag (*Bgl*II) R”. Predicted sizes: *TLOα1* = 2110 bp; *TLO*β*2* = 2180 bp; *TLOγ11* = 1870 bp. (C) PCR to detect reintegration of P_{TET1}-*TLOs* at the *ADH1* locus using primers “ADH1 locus F” and “carTA R”. Predicted sizes = 4451 bp.

1. Staib P, Moran GP, Sullivan DJ, Coleman DC, Morschhauser J. Isogenic strain construction and gene targeting in *Candida dubliniensis*. J Bacteriol. 2001;183: 2859-2865. doi: 10.1128/JB.183.9.2859-2865.2001.
2. Park YN, Morschhauser J. Tetracycline-inducible gene expression and gene deletion in *Candida albicans*. Eukaryot Cell. 2005;4:1328-1342. doi: 10.1128/EC.4.8.1328-1342.2005.
