# Supplementary material for: Deletion of the Candida albicans TLO gene family using CRISPR-Cas9 mutagenesis allows characterisation of functional differences in α-, β- and γ- TLO gene function
Source: PLoS Genet. 2023 Dec 4;19(12):e1011082. doi: 10.1371/journal.pgen.1011082 (PMC10721199; doi:10.1371/journal.pgen.1011082)
Supplement: S8 Fig — (PDF) [file pgen.1011082.s009.pdf]

Figure S8

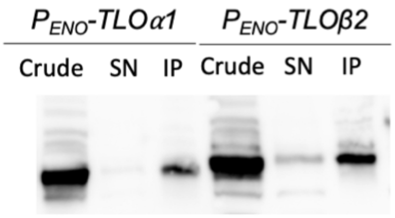

Figure S8. Analysis of anti-HA immunoprecipitation of HA-tagged Tlo proteins. IP of Tlo1-3xHA and Tlo2-3xHA was performed and the level of HA tagged protein analysed by Western blot analysis to determine the relative quantities of Tlo-HA protein in the crude extract, IP supernatants (SN) and in the IP fraction associated with anti-HA beads.
