# Supplementary material for: Deletion of the Candida albicans TLO gene family using CRISPR-Cas9 mutagenesis allows characterisation of functional differences in α-, β- and γ- TLO gene function
Source: PLoS Genet. 2023 Dec 4;19(12):e1011082. doi: 10.1371/journal.pgen.1011082 (PMC10721199; doi:10.1371/journal.pgen.1011082)
Supplement: S9 Fig — (PDF) [file pgen.1011082.s010.pdf]

Figure S9

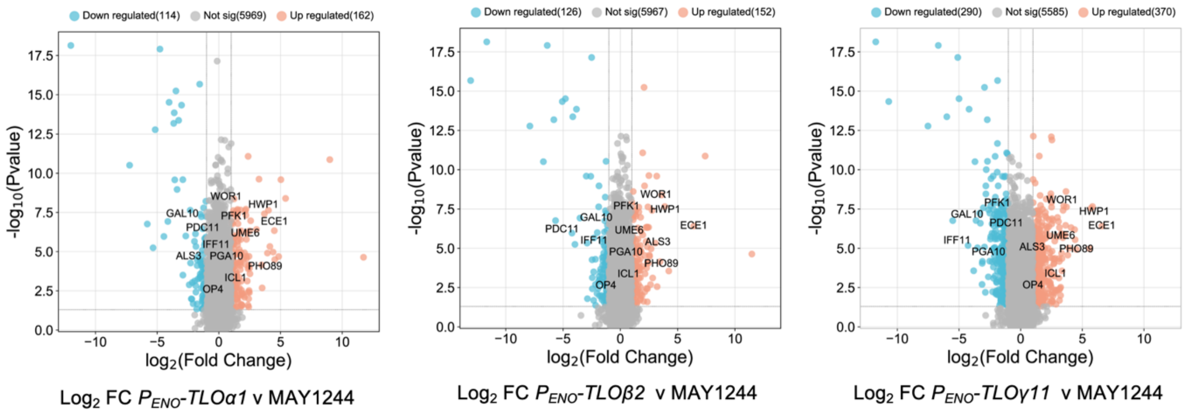

Figure S9. Gene expression in *P_{ENO}-TLO* complemented strains relative to the WT strain MAY1244. Volcano plots show spots representing genes with 2-fold or more change in gene expression (blue decreased; orange increased) relative to MAY1244 (FDR q <0.05).
