# Supplementary material for: Deletion of the Candida albicans TLO gene family using CRISPR-Cas9 mutagenesis allows characterisation of functional differences in α-, β- and γ- TLO gene function
Source: PLoS Genet. 2023 Dec 4;19(12):e1011082. doi: 10.1371/journal.pgen.1011082 (PMC10721199; doi:10.1371/journal.pgen.1011082)
Supplement: S10 Fig — (PDF) [file pgen.1011082.s011.pdf]

Figure S10

A

Regions enriched for Tlo proteins

	Tloα1	Tloβ2	Tloγ11
Repeat Regions	35	66	67
ORF Body	477	2,111	2306
Promoter (-500bp)	179	876	988
ORF/Promoter	552	2,329	2521

B

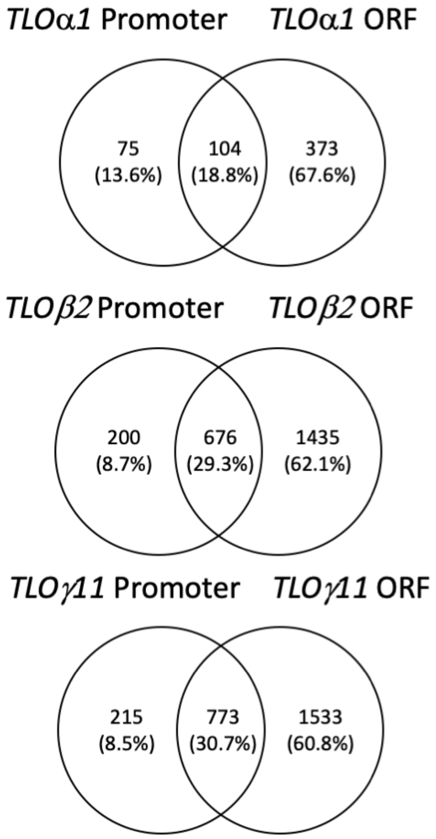

C

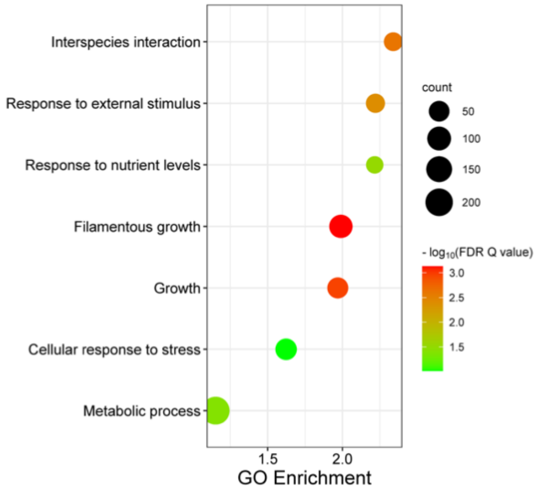

Figure S10. ChIP analysis of HA tagged Tlo proteins. (A) Break down of genomic regions enriched for the indicated Tlo proteins including repeat regions, ORFs, promoters (-1 to -500) and genes where either the ORF or promoter is enriched (FDR q < 0.05). (B) Venn diagrams illustrating the number of genes for each Tlo with enrichments at ORF bodies, promoters or overlapping both regions (C) Results of GO Term analysis of the common set of genes exhibiting significant enrichment (FDR q < 0.05) following ChIP with HA-tagged Tloα1, Tloβ2 and Tloγ11 proteins (n=275). GO analysis was carried out using the GO tool at *Candida* genome Database.
