# Supplementary material for: Deletion of the Candida albicans TLO gene family using CRISPR-Cas9 mutagenesis allows characterisation of functional differences in α-, β- and γ- TLO gene function
Source: PLoS Genet. 2023 Dec 4;19(12):e1011082. doi: 10.1371/journal.pgen.1011082 (PMC10721199; doi:10.1371/journal.pgen.1011082)
Supplement: S11 Fig — (PDF) [file pgen.1011082.s012.pdf]

Figure S11

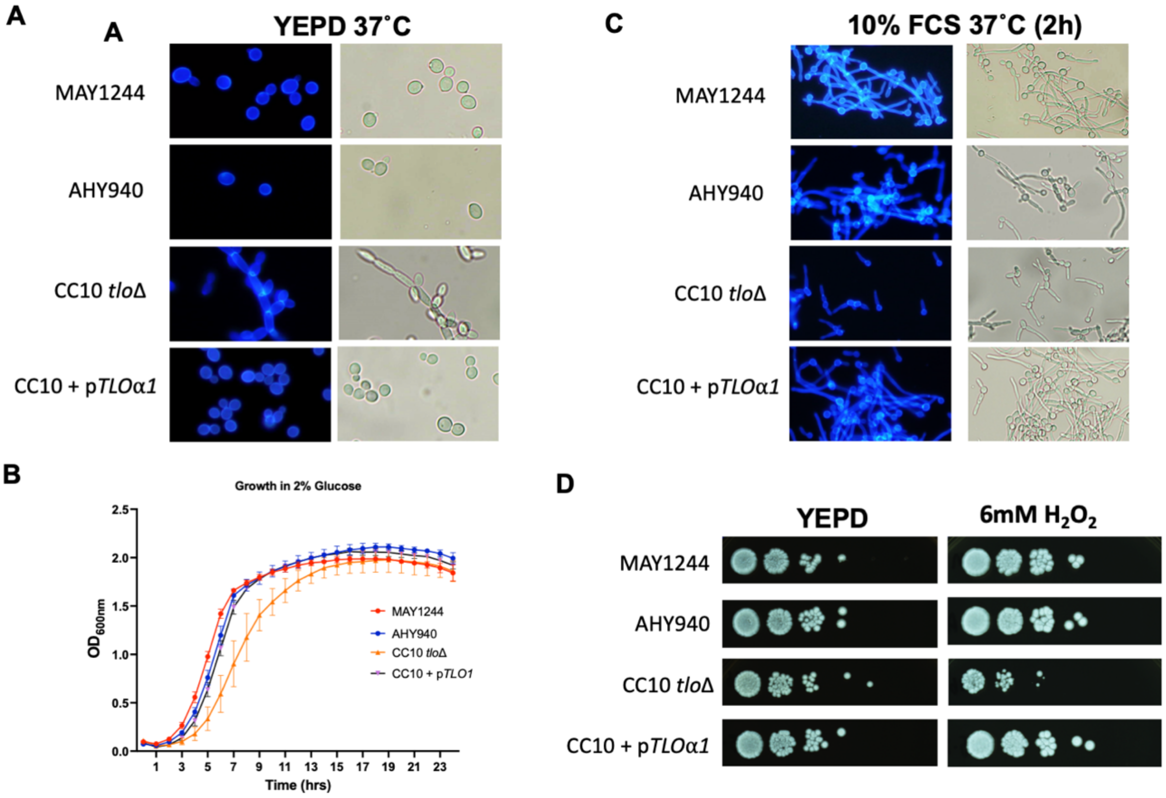

Figure S11. Phenotypic complementation in the CC10 *tlo*Δ mutant by *TLOα1*. (A) Cellular morphologies in YEPD broth at 37˚C. (B) Growth curves generated from the indicated strains following incubation in YEPD plus 2% glucose. Growth was determined by measuring $OD600_{nm}$ in cultures incubated at 37˚C with shaking at 200 rpm. (C) Cellular morphologies following 2h incubation in YEPD + 10% fetal calf serum (FCS) at 37˚. (D) Growth of the indicated strains on YEPD medium supplemented 6 mM H_20_2.
