# Supplementary material for: Deletion of the Candida albicans TLO gene family using CRISPR-Cas9 mutagenesis allows characterisation of functional differences in α-, β- and γ- TLO gene function
Source: PLoS Genet. 2023 Dec 4;19(12):e1011082. doi: 10.1371/journal.pgen.1011082 (PMC10721199; doi:10.1371/journal.pgen.1011082)
Supplement: S12 Fig — (PDF) [file pgen.1011082.s013.pdf]

Figure S12

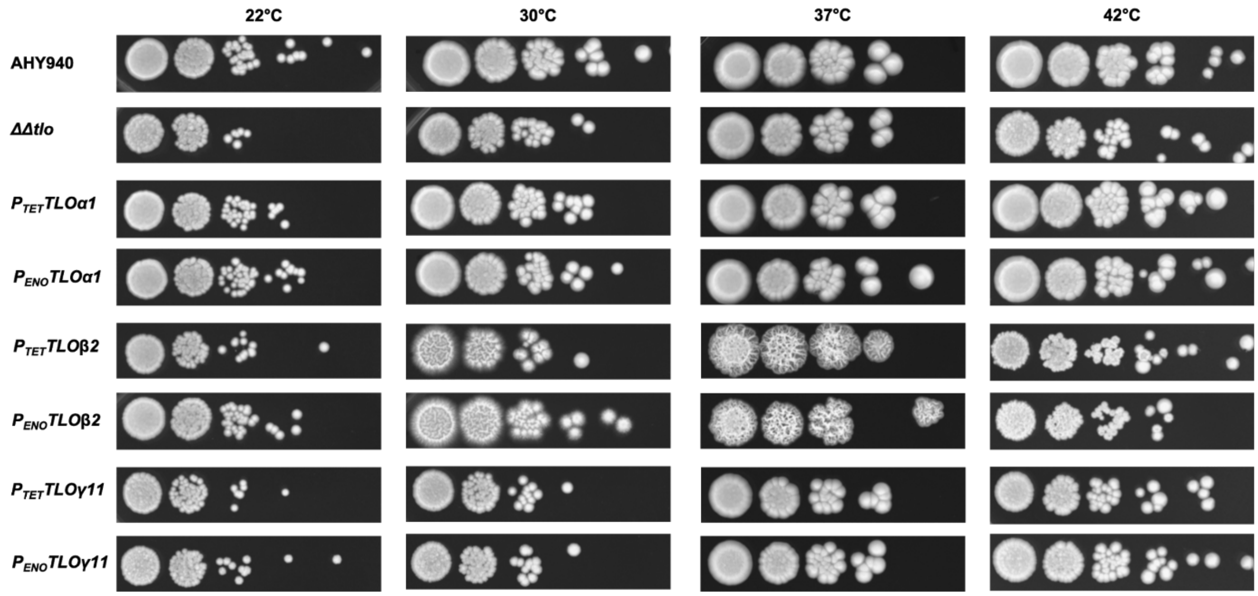

Figure S12. Morphology of *TLO* complemented strains on YEPD agar. The indicated strains were cultured at 22˚C, 30˚C, 37˚C and 42˚C on YEPD agar for 48 h for analysis of colony morphology.
