# Supplementary material for: Deletion of the Candida albicans TLO gene family using CRISPR-Cas9 mutagenesis allows characterisation of functional differences in α-, β- and γ- TLO gene function
Source: PLoS Genet. 2023 Dec 4;19(12):e1011082. doi: 10.1371/journal.pgen.1011082 (PMC10721199; doi:10.1371/journal.pgen.1011082)
Supplement: S14 Fig — (PDF) [file pgen.1011082.s015.pdf]

Figure S14

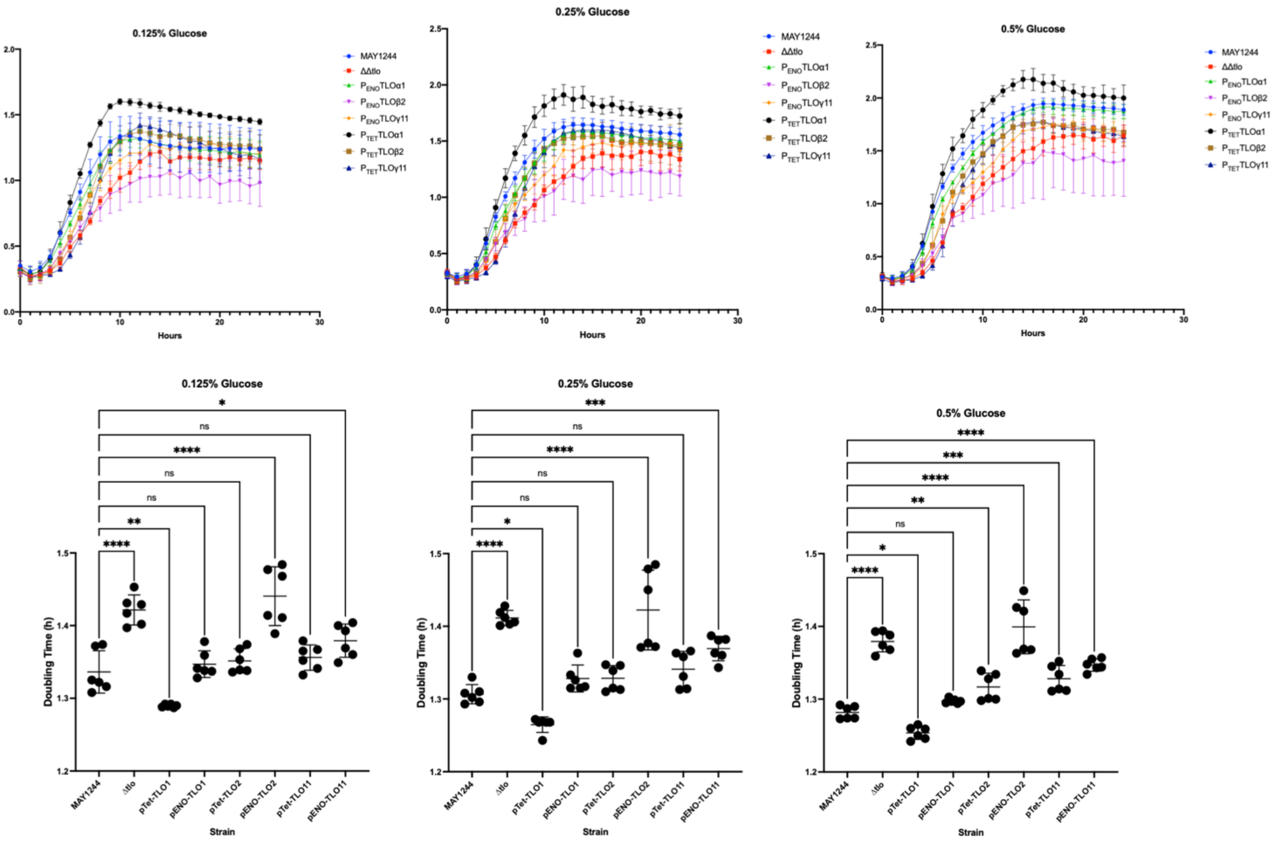

Figure S14. Analysis of growth rates in YEP-glucose at 37°C at 200 rpm (A) Growth curves generated in automated FLUOstar Omega plate reader (BMG Labtech) showing average data and standard deviations from 6 replicate wells. (B) Statistical analysis of data from strains growing in YEPD. Doubling time of each strain is represented by horizontal line (hours), with error bars representing standard deviation and symbols representing each of six replicates. A one-way ANOVA was performed to determine if results were significantly different and a Dunnett's multiple comparisons test was performed to determine which means were significantly different from WT MAY1244 (****= $p < 0.0001$; ***= $p < 0.001$; **= $p < 0.01$, * = $p < 0.05$).
