# Supplementary material for: Deletion of the Candida albicans TLO gene family using CRISPR-Cas9 mutagenesis allows characterisation of functional differences in α-, β- and γ- TLO gene function
Source: PLoS Genet. 2023 Dec 4;19(12):e1011082. doi: 10.1371/journal.pgen.1011082 (PMC10721199; doi:10.1371/journal.pgen.1011082)
Supplement: S15 Fig — (PDF) [file pgen.1011082.s016.pdf]

**Figure S15**

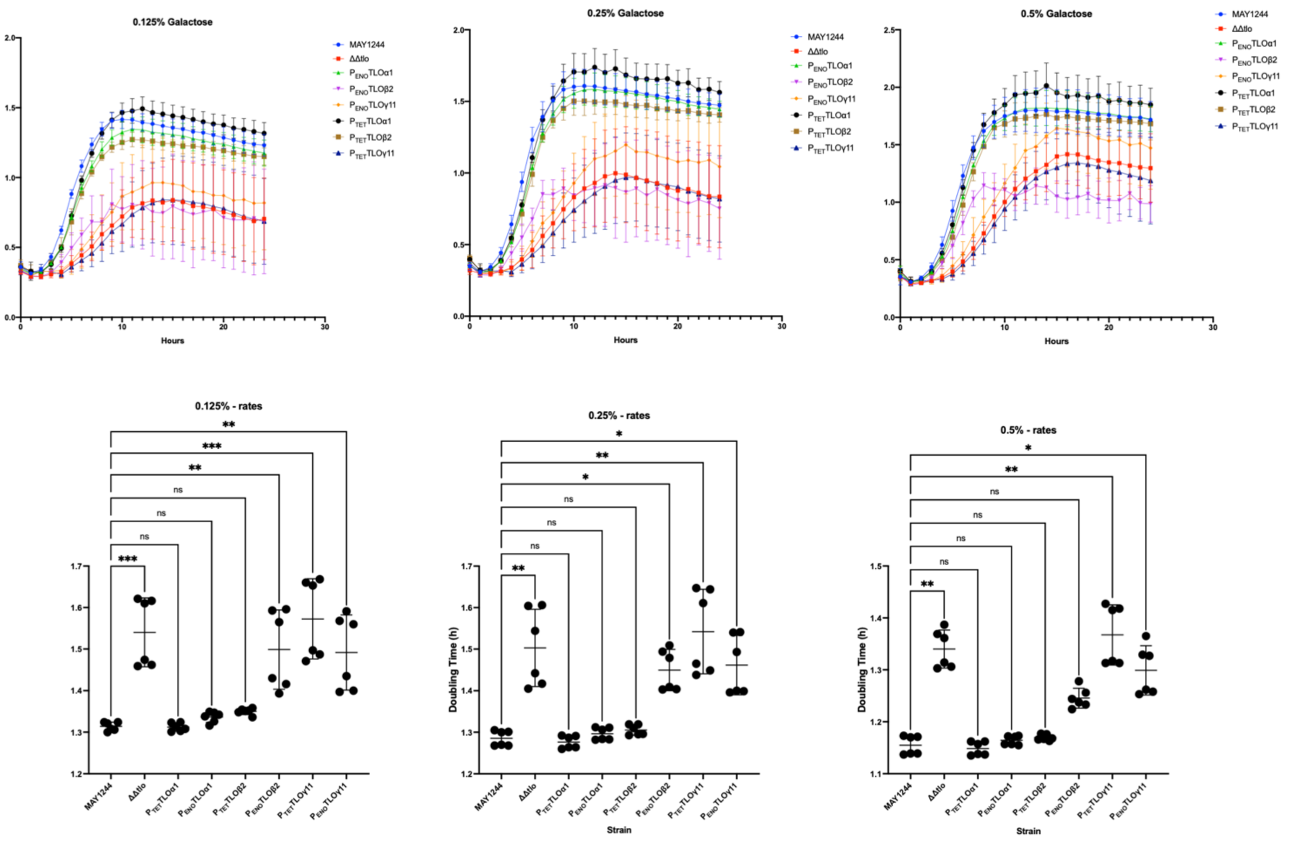


**Figure S15. Analysis of growth rates in YEP-galactose at 37°C at 200 rpm**. (A) Growth curves generated in automated FLUOstar Omega plate reader (BMG Labtech) showing average data and standard deviations from 6 replicate wells. (B) Statistical analysis of data from strains growing in YEP-gal. Doubling time of each strain is represented by horizontal line (hours), with error bars representing standard deviation and symbols representing each of six replicates. A one-way ANOVA was performed to determine if results were significantly different and a Dunnett's multiple comparisons test was performed to determine which means were significantly different from WT MAY1244 (****= $p < 0.0001$; ***= $p < 0.001$; **= $p < 0.01$, * = $p < 0.05$).
