# Supplementary material for: Deletion of the Candida albicans TLO gene family using CRISPR-Cas9 mutagenesis allows characterisation of functional differences in α-, β- and γ- TLO gene function
Source: PLoS Genet. 2023 Dec 4;19(12):e1011082. doi: 10.1371/journal.pgen.1011082 (PMC10721199; doi:10.1371/journal.pgen.1011082)
Supplement: S16 Fig — (PDF) [file pgen.1011082.s017.pdf]

**Figure S16**

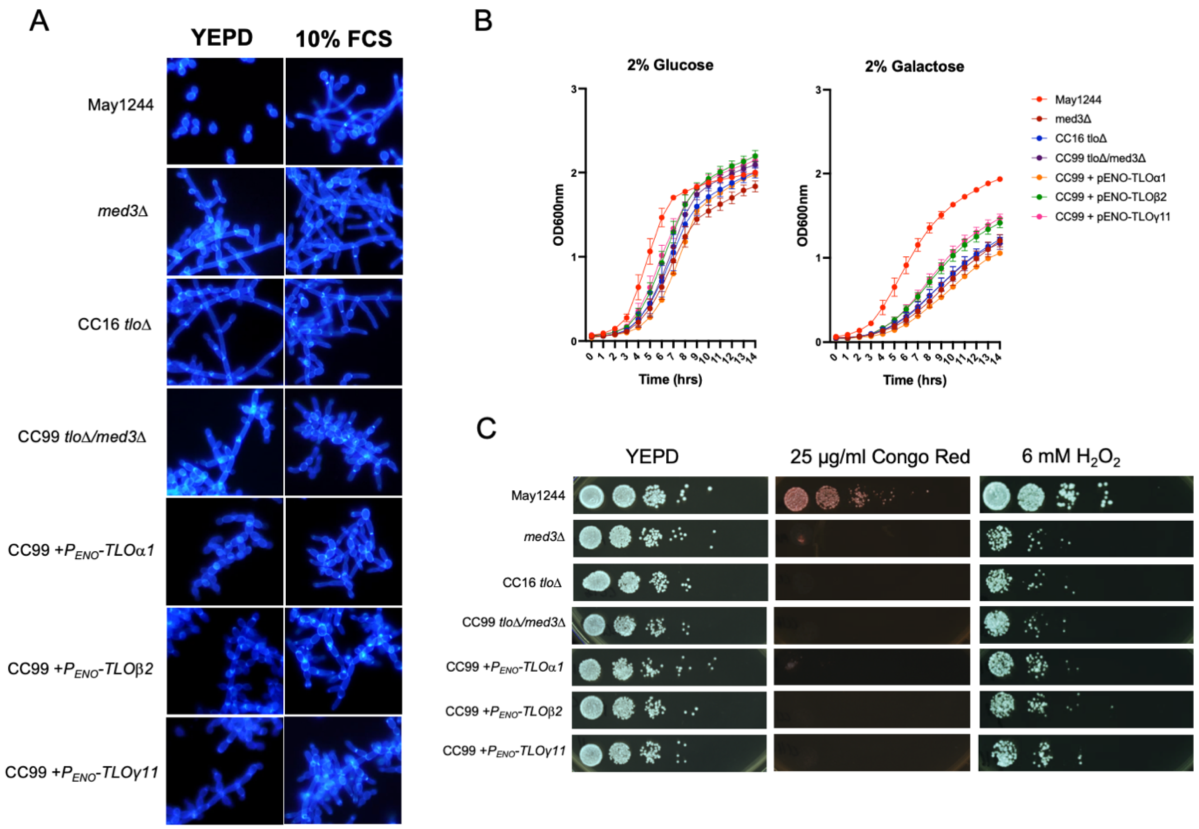


**Figure S16. Comparative phenotypic analysis of a *tlo*Δ/*med3* double mutant (CC99) complemented with *TLOα1*, *TLO*β*2* and *TLO*γ*11*.** (A) Cellular morphologies in YEPD broth and following 2h incubation in YEPD + 10% fetal calf serum (10% FCS) at 37˚C observed following calcofluor white staining. (B) Growth curves generated from the indicated strains following incubation in YEPD plus 2% glucose or 2% galactose, as indicated. Growth rates were determined by measuring $OD600_{nm}$ in cultures incubated at 37˚C with shaking at 200 rpm. (C) Growth of the indicated strains on YEPD medium supplemented with Congo Red (25 μg/ml) or $H_2O_2$ (6 mM).
