# Supplementary material for: Deletion of the Candida albicans TLO gene family using CRISPR-Cas9 mutagenesis allows characterisation of functional differences in α-, β- and γ- TLO gene function
Source: PLoS Genet. 2023 Dec 4;19(12):e1011082. doi: 10.1371/journal.pgen.1011082 (PMC10721199; doi:10.1371/journal.pgen.1011082)
Supplement: Attachment — Submitted filename: Response to reviewers.docx [file pgen.1011082.s034.docx]

**RE: MS PGENETICS-D-23-00629 Response to Reviewers’ Comments**

Dear Dr. Forche

We thank you and the three reviewers for reviewing our manuscript PGENETICS-D-23-00629. We are pleased that it seems that all three reviewers found merit in our study and that the data presented are “*impressive and important*” (Reviewer #1) and “*significant and well-presented*” (Reviewer #2). We are particularly grateful for the time you and the reviewers have taken in providing such thorough and comprehensive comments. This study represents the first time that this large gene family has been investigated using gene deletion in *C. albicans*. We have confirmed that Med2/Tlo plays a role in the control of a wide range of cellular activities and we clearly show that there is evidence for functional diversity between the three Tlo clades, in particular in relation to the functionality of γ-*TLO* genes. The data supporting these findings are extensive, resulting in a total of 27 figures, and represent the first stage in dissecting how the Tlos exert their diverse roles.

We thank the reviewers for their many suggestions and for recommending some very helpful additional experiments. In addition, we are grateful to you for agreeing to an extension for our resubmission thus providing us with the opportunity to perform these experiments. Wherever feasible, we have taken on board as many of the reviewers’ recommendations as possible and have revised the manuscript accordingly. This has resulted in significant alterations to the original text and figures. In order to facilitate you in seeing where these changes have been made we have uploaded a supplementary document (entitled “Marked copy”) where specific text addressing individual comments has been highlighted using the Comments function of Microsoft Word. If you would like a version of the document showing all of the textual changes (using Track Changes) we can of course also provide this if required.

Our responses to each of the reviewers’ points raised are listed in order overleaf. We hope that you agree that by addressing the reviewers’ comments and recommendations we have significantly improved the manuscript and that it is now acceptable for publication.

Yours sincerely,

Gary Moran	Derek Sullivan

**Responses to Reviewers’ Comments**

**Reviewer 1**

This referee has provided a very thorough critique of our manuscript and clearly understands the importance of this “*long sought after goal*” and the difficulties we faced in deleting such a large and closely related gene family. We agree that the role of the *TLO* family in so many cellular functions represents a strength and a weakness and that perhaps this manuscript represents the beginning of a thorough evaluation of this complex gene family, rather than the end. We particularly appreciate the reviewer’s suggestion that “*it is (not) practical to ask the authors to completely reconfigure, but need to address whether the deletion of the TLOs, or any of the restoration of Tlos, is originating from the reconstitution of a stable functional Tail module of Mediator, or a role of the Tlo beyond being a direct member of the Tail module*”. We hope that our responses to the referees’ comments below go some way to try to address this question.

Our responses to each of the numbered points are as follows:

1. **Reviewer comment:** Figure 1 could benefit from some greater context. It would be helpful to compare the impact of the *tlo* deletion in *C. albicans* versus the Med3 mutant that they made.

Response: We agree that additional context would be useful here. We have now included reference in the text to comparisons between Mediator tail mutants we have generated in *C. dubliniensis* (*i.e. tloΔ* and *med3Δ*) and previously published *C. albicans med7Δ* mutant data. These data bear many similarities to our *C. albicans tloΔ* data with regards to reduced expression of genes involved in carbohydrate metabolism, amino acid metabolism and increased expression of genes involved in ribosome biogenesis (lines 200-203). We compare the phenotypes of the *tloΔ* and *med3Δ* mutants throughout the text and in the discussion (lines 660-662). The most important difference between *CatloΔ* and previous studies of Mediator is the apparent unique role of CaTlo in the regulation of expression of opaque phase specific genes and this is now highlighted (lines 204-206).

2. **Reviewer comment**: Also related to Figure 1, I could not find whether there was an analysis of the correlation between regions with lower RNAP II enrichment and the genes whose expression were impacted.

Response: We have performed additional analysis of these data and have now included this in Figure S5. Essentially, genes not bound by RNAPII in the tlo*Δ* mutant show a reduction in raw mRNA expression levels (lines 220-223).

3. **Reviewer comment:** A comparison of the amount of the tagged Tlo protein to the amount of a comparable Mediator subunit such as a tagged Med3 subunit or a tagged Tlo protein in the parent strain would help determine whether the amount of the restored Tlo is sub-stoichiometric, stoichiometric, or super-stoichiometric with regards to the other subunits of Mediator.

Response: We agree that determining the amount of tagged Tlo protein relative to Mediator would be informative. To address this we have generated some additional strains including a wild-type strain with HA-tagged Med3 and a derivative of the Tlo null strain that expresses both *pENO-TLO1*-HA and HA-tagged Med3 (lines 931-935). Western blots show that expression of *TLO* from both *p_TET_* and *p_ENO_* promoters generates levels of Tlo protein in excess of Med3. This ranges from ~5-fold excess in the pTet strain to ~30-fold excess in the *p_ENO_* strains (see new panel (E) in Fig. 3 (lines 333-335) and lines 315-322). This additional information raises some interesting questions, particularly surrounding Tloβ2. Tloβ2-expressing strains exhibit increased filamentous growth and this phenotype is enhanced when expression is increased from the *p_ENO_* promoter (Fig. 6B). This increased Tloβ2 protein is therefore likely to be in excess of Mediator and must be exerting these effects in a Mediator-independent fashion. We discuss the significance of this and relate these data to similar effects described for *C. dubliniensis* Tlo2 (lines 690-699).

4. **Reviewer comment:** It is concerning that in Figure 3, that there are no other Mediator subunits other than the two other tail subunits in the mass spec experiments on the pull downs.

Response: In order to demonstrate the efficiency of the anti-HA pull down, we have now included an additional figure (Fig S8) showing the amount of HA tagged protein in the IP fraction and supernatant. It appears to us that the pull-down experiments are working efficiently and that very little HA-tagged protein remains in the supernatant (lines 299-300). It seems unlikely that poor efficiency of the IP is the reason why no other Mediator subunits have been recovered.

Previous studies where *Candida* Mediator has been purified in its entirety have generally involved IP of head subunits (e.g. Med8) which may have more and/or stronger protein-protein interactions resulting in pull-down of more proteins from the complex. Here, we have tagged Tlo/Med2 which is a relatively peripheral subunit and may explain why we have only recovered other tail subunits. The purpose of the experiment was to verify Mediator incorporation and we suggest that these data support this.

We were encouraged to detect interaction with Rpc10 (Rpb12), thereby linking Tlo/Med2 with RNAP II. Although the reviewer cites this as unusual, interactions between Mediator tail and Rpc10 have been previously described (Lim et al, PMID: 17919657). Rpc10 was consistently detected *in all* Tlo pull down experiments with good coverage.

The reviewer is correct to point out that some of the more unusual interactions will require verification. For example Tlo is unlikely to have a direct functional interaction with translation proteins; their detection may be a result of isolating the protein during or soon after translation by the ribosome. We have added this possibility to lines 710-712.

5. **Reviewer comment:** How do the restorations compare to the parent strain?

Response: We have added a new supplementary figure (Fig. S9) which shows the results from comparing the transcriptomes of the reintegrants to that of the wild-type strain. *TLO1* and *TLO2* largely restore a transcriptome similar to wild-type, although not all genes return to a wild-type level of expression. For example, some hypha-specific genes remain upregulated in both reintegrants. This is shown in the new Fig. S9 and described in lines 362-367.

6. **Reviewer comment:** This could be done by restoring a Tlo with just the Med2 domain

Response: We have not yet carried out functional analysis of the N- and C- terminal Tlo domains. Previous studies have shown that deletion of the Med2 domain prevents Mediator incorporation and deletion of the C-terminal activating domain abrogates transcriptional activating activity. It is likely that neither of these truncated proteins would complement any of the phenotypes in the *tloΔ* null mutant however, this is certainly something we will consider in our future work.

7. **Reviewer comment:** by tagging one or more other Mediator subunits and assessing their localization. In the absence of this it is hard to interpret the significance of the Tlo localization.

Response: This would certainly be an interesting experiment and could help determine if free Tlo binds to the chromosome at sites independently of Mediator. This is beyond the remit of the current study and could be included in a future study investigating the presence and/or role of putative "Mediator-free” Tlo.

8. **Reviewer comment:** do any of the restoration properties of the Tlos depend on an intact tail module? This could be done by making a med3 deletion in the tlo-delta background.

Response: We agree that this is an interesting experiment which we had considered as part of a future study. The fact that the single *med3Δ* and *tloΔ* mutants have similar phenotypes suggests that the functionality of the Tlo proteins requires them to be anchored to Mediator via Med3. However, to comply with as many referee suggestions as possible we have, in response to this comment, made a double *tloΔ/med3Δ* mutant and expressed the three clade-representative *TLO* genes in this background. As expected, under the phenotypic conditions tested we saw that expressing the reintegrated *TLO* genes had no effect on the phenotype of the double mutant, which phenocopied the original *tloΔ* and *med3Δ* mutants. This suggests that, at least under the conditions tested, the Tlo proteins must be anchored to Mediator (via Med3) to exert their effect. These data are described in lines 616-627 and a new supplementary figure (Fig. S16) and the implications of these data are discussed in lines 682-686.

9. **Reviewer comment:** It is unclear whether the difference in the impact of the Tlo-alpha versus the Tlo-beta is an issue of sequence or of expression level

Response: We agree that expression level of each Tlo may impact on phenotype. Our interpretation of the Western blot in Fig. 3B was that expression of P_TET_Tloα1 and P_TET_Tloβ*2* were broadly similar based on comparisons to the PolII loading control. We have now carried out densitometry of the original images and can report that relative to the PolII control, the P_TET_ and P_ENO_ Tloα1 and Tloβ2 constructs have comparable expression levels. These normalised expression levels are now included in Fig. 3B (line 328-329) and described in lines 287-289. These new data support the idea that the differences in glycolytic gene expression between the P_TET_Tloα1 and P_TET_Tloβ*2* reintegrants (Fig. 7E) is based on functional differences rather than expression levels (see lines 509-511). However, we agree that the gene expression patterns in strains with the more highly expressed P_ENO_Tloα1 and P_ENO_Tloβ*2* constructs are more similar, indicating that the lesser functionality of P_TET_Tloβ*2* can be rescued by increasing expression (lines 509-511). Further evidence for different functionality between Tloα1 and Tloβ*2* comes from their roles in regulating hypha-specific genes, where increasing Tloβ*2* expression results in increased expression of hypha-specific genes, which is not seen in Tloα1 expressing strains (Fig 4C, lines 357-361). To make these expression related changes clearer, we have reconfigured the heatmaps in Fig. 7E and Fig. 4C to directly reflect expression relative to the WT strain (in the previous heatmaps the expression changes were scaled according to each gene).

**Smaller point**

1. Reviewer comment: The inability of the tlo-delta strain to switch is likely a result of a non-functional Mediator Tail.

Response: We agree, we have amended the text accordingly (line 208).

2. Reviewer comment: It is not all that remarkable that transcription factors were 'notably absent' from the Tlo pull down mass spec experiments

Response: We agree, we have amended the text accordingly (line 310).

**Reviewer #2**

We are grateful to the reviewer for stating that we presented “*a well-written and thorough manuscript*” and that the “*the generation of a 14-gene deletion strain and the subsequent karyotyping and genomic analysis is significant and well presented*”. We appreciate that there are “*a few concerns that temper enthusiasm for what is otherwise a thorough and well-presented manuscript*” and we have attempted to address the issues raised below:

1. **Reviewer comment:** While the authors do comment on the differences in expression, they largely ignore these differences in their analysis and discussion of the various assays which rely upon these strains. While there are clearly some differences in the phenotypes of the Tloα and Tloβ complemented strains, even when comparing the variants that have similar Tlo protein expression levels, this potential caveat to the presented work is not adequately discussed.

Response: Expression level is an important factor in our data and we have carried out several revisions to the text to emphasise this. In response to Reviewer #1 (Comment 9), we have carried out image densitometry analysis on Western blots showing that relative to RNAP II levels, the P_TET_ and P_ENO_ constructs of Tloα1 and Tloβ2 have similar expression levels (Fig. 3B, lines 287-289). As detailed below in response to Comment 7, this analysis supports the notion that the differences in glycolytic gene expression observed between P_TET_Tloα1 and P_TET_Tloβ*2* reintegrants are based on function rather than expression. However, we also accept that this difference can be rescued by increased Tloβ2 expression and we have added an additional sentence stating that the “increased expression of *TLOβ2* from the *ENO1* promoter resulted in increased levels of glycolytic gene expression which were more equivalent to that observed in Tloα1 reintegrant strains (Fig. 7E)” (lines 509-511). To make these expression related changes clearer, we have reconfigured the heatmaps in Fig. 7E and Fig. 4C to directly reflect expression relative to the WT strain (in the previous heatmaps the expression changes were scaled according to each gene).

The filamentous growth phenotype exhibited by the Tloβ2 reintegrants also exhibited an expression related component, with the P_ENO-_Tloβ2 reintegrants exhibiting increased filamentous growth in YPD (Fig. 6B) and increased hypha-specific gene expression (Fig. 4C and lines 357-361) which was not observed in Tloα1 reintegrants and this is now noted in the text (lines 434-435).

However, some other Tloβ2 phenotypes cannot be rescued by increased expression, including the reduced capacity to restore tolerance to tBOOH, now highlighted in lines 537-539.

These expression related differences are referenced in the discussion in lines 740-743.

2. **Reviewer comment:** ChIP data showing distinct but overlapping binding “is quite possibly an artifact of the extremely low protein expression level of Tloγ, which is likely to lead to false positive peaks of “enrichment” at highly repetitive or highly expressed genomic loci”

Response: See response to comment 6 below. We have removed most references to repeat sequence-binding as these finding require further validation and rewritten the relevant section of the results and discussion (see lines 763-76) to reflect the referee’s recommendation.

*Additional/detailed comments:*

3. **Reviewer comment:** The authors need to comment on the size cut-off that is used to delineate a "large LOH"

Response: We have removed reference to the scale of LOH (see line 160)

4. **Reviewer comment:** It would be helpful to know how many colonies they had to screen to get the full tlo knockout strains (i.e. deletion efficiency).

Comment: We have now amended the text to include this information (see Line 837-839).

5. **Reviewer comment:** In the ChIP assay the authors use "Gene" to designate regions including ORFs + 500bps upstream, which they indicate harbor most of the Tlo protein enrichment. However, this designation seems somewhat arbitrary….. It would be helpful if the authors could present a more thorough analysis of Tlo protein localization.

Response: There is sound logic to our examination of the ORF and the -500 upstream region. In our previous Tlo ChIP study (Haran et al. PLoS Genet 10(10): e1004658. doi:10.1371/journal.pgen.1004658) we show that the majority of Tlo binds to ORFs and that upstream binding is predominantly within ~400 bp of the ORF start site ( see Haran et al. Fig S9). This is also true of Mediator in S. cerevisiae (Eyboulet et al, NAR, doi: 10.1093/nar/gkv782). We don’t know the significance of binding outside these regions. This may be non-Mediator associated Tlo, but further ChIP experiments would be necessary to determine this and comment on its significance.

In response to the request for more analysis, we elaborate in more detail on the proportion of Tlo binding at ORFs and at promoters (lines 383-388 and Fig. S10). We also present some additional analysis on the significance of Tlo localisation to gene expression, whereby we show Tlo binding is associated with increased expression of those specific genes in the reintegrant experiments (lines 395-400 and Fig. 5C (line 407)). We have also now included the scale information for the X-axis in Fig. 5B as requested.

6. **Reviewer comment:** It is also very likely that the low level of overall enrichment that would be expected from poorly expressed proteins (i.e. Tloγ11-HA) may have led to overamplification of repetitive/highly expressed sequence elements in the genome, while the increased enrichment (at non-repetitive loci) observed with a more abundant protein may have reduced the amount of unintended amplification of these sequences. ….The addition of an input DNA control and/or mock IP sample would enable the authors to directly assess this question and determine more definitively what loci are truly occupied by which Tlo proteins.

Response: We have clarified in the Materials and Methods that an input DNA control was used (Line 995-996; no selection by immunoprecipitation). However, we accept the referee’s suggestion that the chromosomal localisation of Tloy11 in the ChIP experiments may be a reflection of the low level of expression of the protein (see lines 763-765). Further analysis would be required to verify the binding to repeats. We have decided to remove the panels in Fig. 5 referring to binding to telomeres and MRS regions. We have significantly altered the text of this section and removed most of the references to repeat binding.

7. **Reviewer comment:** On lines 478-480 the authors state that *TLOα1* is a stronger activator of glycolytic gene expression compared to *TLO*β*2*, most notably when comparing the pTET expressed versions…..The authors should comment on the relative expression levels of the Tlo proteins under these different promoters and how that might influence the interpretation of their observations in this section of the manuscript. Also not clear which promoters are being referred to in Fig. 7D

Response: Fig 7D refers to the P_TET_ versions and this has now been highlighted in the legend. In response to this comment and comments made by reviewer 1, we have quantified expression of the Tlo proteins in Fig. 3B relative to the loading control (RNAP II). As now shown in Fig3B, relative to the RNAP II control, the P_TET_ and P_ENO_ constructs of Tloα1 and Tloβ2 are largely equivalent (Line 287-289). As stated in response to Reviewer 1, these new data support the hypothesis that the increased functionality of P_TET_-Tloα1 in Fig 7D & E is not solely expression related. However, we do accept that increasing expression of Tloβ2 using the *ENO1* promoter rescues some of the reduced functionality, and this is now noted in the text (lines 506-508) and discussed (lines 740-743). We have also changed the heatmap presentation in Fig 4 and Fig 7 to better compare the differences between the P_TET_ and P_ENO_ constructs relative to WT).

8. **Reviewer comment:** Lines 495-496. These genes were added back individually, not in tandem, and there were at least two combinations of complemented gene and stressor that did not yield wild-type tolerance. This sentence should be edited accordingly.

Response: We have clarified that each gene was added back individually (line 524-535) and removed reference to the WT in the following line.

9. **Reviewer comment**: The sentence beginning on line 501 refers to GSEA enrichment scores and refers the reader to Figure 4A, but no GSEA enrichment scoring is presented in that figure. The addition of a supplemental figure to directly present the referred to GSEA enrichment scores across the different complemented strains would be helpful.

Response: This should have referred to Fig 4B, which shows the XS(2006)_UP GSEA data. This has now been corrected (line 532). The results of the GSEA analysis have now also been included in supplementary Tables S10-S13.

10. **Reviewer comment:** The sequence of genotypes, along with the orientation of the bar charts, is different between figure 9A and 9B, which makes it confusing to compare these two panels.

Response: The genotypes in Fig 9(A) have been reordered (P_TET_ before P_ENO_) and both graphs are now in the vertical orientation as recommended by the referee (line 584).

11. **Reviewer comment:** Line 708 refers to the “highly expressed PENO-TLOϒ11 gene”, however the protein expressed from this construct could only be detected by mass spectrometry and did not appear on a western blot that easily detected the alpha and beta variants. As such, this statement could easily be misconstrued by a reader that did not closely evaluate the western blot shown in figure 3B.

Response: This sentence has now been deleted in response to Referee 3’s recommendation to reduce the length of the Discussion.

12. **Reviewer comment:** Line 141-142 the authors state that Med3 is required for Tlo incorporation into the Mediator complex, and present data indicating that the strains lacking all *TLO* genes have reduced virulence in the *G. mellonella model*, yet do not discuss or contextualize the observation that the med3 deletion strain phenocopies the wild-type strain in this model. This result should be addressed and discussed.

Response: We thank the referee for bringing this to our attention. Due to an oversight incorrect data were presented in the original submission. We have now included the correct data in Figure 2 which shows that the med3Δ mutant phenocopies the tloΔ mutant in all respects, including the *Galleria* infection model (panel F, line 267).

**Reviewer #3**

We appreciate the referee’s comments concerning the “*abundant amount of supportive data*” and the rigorously performed experiments. We agree that we may not have answered the question as to why the TLO genes have undergone such dramatic expansion, but we believe that we have now established the important role of the Tlo family in numerous cellular functions as well as confirming the functional diversity between the three clades.

1. **Reviewer comment:** The Discussion is entirely too long and is mostly a repetition of the results with little insight as to their interpretation. It needs to be cut in half and focused on why various family members have altered phenotypes, TLO-related mechanisms that may allow for fine tuning of virulence and commensalism, etc.

Response: We have reduced the length the Discussion as recommended by the referee. However, as we have performed some additional experiments (e.g. expression of reintegrated TLO genes in the double *tloΔ/med3Δ* mutant, HA tagging of Med3) this has necessitated additional text, however, we have attempted to keep this to a minimum. Despite this, the current discussion is approximately 10% shorter than the original submission.

2. **Reviewer comment:** In constructing *tloΔ*, the authors realize that such a large number of double strand breaks may lead to unwanted chromosomal aberrations……More comments of this (rearrangements) in the discussion and alternative approaches may be warranted.

Response: We have added some additional text in the discussion describing a strategy which may reduce the likelihood of chromosomal rearrangements by first of all deleting the TLO34 gene which seems to be a hotspot for translocations (line 656-657)

3. **Reviewer comment:** Did the authors perform transcriptional profiling on the *med3Δ*? If so, how does this compare to *tloΔ*?

Response: We have not yet carried out this experiment, but we have now compared our data to other Mediator mutants (i.e. *med7Δ* and *med31Δ*) and *C. dubliniensis* *med3Δ* to provide context (lines 200-203). See also Reviewer 1, comment 1.

4. **Reviewer comment:** Fig. 2: It is somewhat surprising that *tloΔ* shows increased filamentous growth at 30C in YPD, but reduced filamentous growth on Spider medium. Can the authors provide some commentary on this disparity in the Discussion?

Response: We believe that these represent two distinct phenotypes. In liquid YPD we refer to the mutant as growing as “pseudohypha-like” chains of cells, which may be due to a cell separation defect rather than filamentous growth. On solid Spider medium the WT, unlike the mutant, produces true hyphae. We have amended the text to clarify this (Line 246-247)

5. **Reviewer comment:** The virulence data (e.g., Fig. 2F) is very modest. What happens at longer time points (e.g., d4)? Do the curves converge?

Response: The timeframe used is the standard Galleria infection protocol used in most labs. The differences shown are reproducible and statistically significant. Extending the infection period usually leads to larval death eventually. Wild-type strains generally result in death within 48 hours and less virulent strains usually cannot replicate this within the same time period.

6. **Reviewer comment:** Given that TLO genes are at subtelomeric regions, how stable is TLO copy number in a WT strain? How much might this impact strain-to-strain phenotypic variation? While this is not addressable in the current study, it could be included in the Discussion.

Response: Whole genome sequencing of 21 clinical *C. albicans* isolates revealed that the TLO family “*is continuing to rapidly evolve, including changes both in the number and relative positions of TLO genes within the genome”.* Hirakawa [22] (2015)

Anderson [23] (2015) have also shown that *subtelomeric recombination is a rapid mechanism of generating genotypic diversity through alterations in the number and sequence of related TLO family members*”. These data suggest that the *TLO* family repertoire can differ between strains and that this may contribute to phenotypic variation. We have expanded on this in the Discussion (Line 632-636).

7. **Reviewer comment:** What does *TLO* family member gene expression look like under various conditions? Have the authors mined publicly available existing RNA-Seq datasets to better grasp which family members are independently or co-ordinately regulated to given stressors?

Response: There are lots of references to altered *TLO* expression in various RNA seq experiments (e.g. Amorim-Vaz et al, [34] cited here Line 747). However, there is a major caveat to using Illumina sequencing technology examine Tlo expression as the gene sequences are so similar that reads are easily mis-assigned to the wrong Tlo gene. QRT-PCR is also problematic as gene-specific primers are difficult to generate. Presently, we are planning to used long-read Nanopore technology to carry out cDNA seq. This has the potential to properly discriminate between Tlo paralogs and will allow us to carry out the experiments the reviewer suggests.

8. **Reviewer comment:** What is the role of the *TLO* family members? They do not appear to control phenotypes similar to the other members tested. Are all *TLO* members similarly non-functional?

Response: We are not sure what the Referee is referring to in this comment. Do they mean the TLOγ family? If so we are still not sure what the role of the Tloγ proteins are. Based on our data from two examples we are convinced that they are certainly clearly distinct from the other two clades. Future work will investigate creating mutants with all clade members deleted, or just comparing the genes from a single clade. We have added some additional text speculating on the possible role of these genes (Lines 791-794). We will be particularly interested in investigating any relationship between the *TLO* genes and recent discoveries concerning RNAi from Alessia Buscaino’s group.

9. **Reviewer comment:**  I caution the authors to carefully go over Figure numbers and callouts in the text. There are multiple errors.

Response: We are grateful to the referee for pointing out these errors which have now been corrected.

*Minor comments*

1. Line 194: do the authors means “gene expressed during hyphal growth…”?

Response: We can see why this may be confusing. As shown in Fig. 1B the Hyphae-DN data set is enriched in the WT compared to the mutant, we have tried to make this clearer by referring in the text to genes “regulated” during hyphal growth.

2. Line 249: should be Fig. S5B

Response: Due to the addition of a new supplementary figure, this is now Fig. S6B (see line 259).

3. Line 252: should be Fig. 2F

Response: This has now been corrected (line 261).

4. Line 488: fix “the_tloΔ”

Response: This has now been corrected (line 489).

5. Line 489: incorrect figure cited

Response: This has now been corrected (line 490).

6. Figure 7: labels on x-axis do not match in panels A and B.

Response: This has now been corrected

7. Line 552: should read “hyhphae”.

Response: This has now been corrected (line 580).

8. Line 947: change to “spheroplasted”

Response: This has now been corrected (line 980).
